# Visual influence on bimanual haptic slant adaptation

**DOI:** 10.1167/jov.24.1.8

**Published:** 2024-01-11

**Authors:** Catharina Glowania, Marc Ernst, Sarah Hanke, Loes van Dam

**Affiliations:** 1 Department of Cognitive Neuroscience, Bielefeld University, Bielefeld, Germany; 2 Department of Applied Cognitive Psychology, Ulm University, Ulm, Germany; 3 Department of Human Sciences, Institute for Psychology/Centre for Cognitive Science, Technical University of Darmstadt (TU Darmstadt), Darmstadt, Germany

**Keywords:** haptic adaptation, vision, haptics, bimanual adaptation, bimodal exploration

## Abstract

Adapting to particular features of a haptic shape, for example, the slant of a surface, affects how a subsequently touched shape is perceived (aftereffect). Previous studies showed that this adaptation is largely based on our proprioceptive sense of hand posture, yet the influence of vision on haptic shape adaptation has been relatively unexplored. Here, using a slant-adaptation paradigm, we investigated whether visual information affects haptic adaptation and, if so, how. To this end, we varied the available visual cues during the adaptation period. This process ranged from providing visual information only about the slant of the surface, or the reference frame in which it is presented, to only providing visual information about the location of the fingertips. Additionally, we tested several combinations of these visual cues. We show that, as soon as the visual information can be used as a spatial reference to link the own fingertip position to the surface slant, haptic adaptation is very much reduced. This result means that, under these viewing conditions, vision dominates touch and is one reason why we do not easily adapt to haptic shape in our daily life, because we usually have visual information about both hand and object available simultaneously.

## Introduction

When touching a slanted surface for a period of time, it will feel less slanted. This mechanism is called adaptation. When next presented with a physical surface that is level, this nonslanted surface will feel as though it was slanted in the opposite direction (adaptation aftereffect). In the past, slant adaptation aftereffects were mainly investigated by adapting some environmental property in a given sense, for instance, the haptic sense in isolation (van Dam, Plaisier, Glowania, & Ernst, 2016; Glowania, Plaisier, Ernst, & Van Dam, 2020), or the visual sense in isolation (e.g., Bergman & Gibson 1959; Balch, Milewski, & Yonas, 1977; Berends, Liu, & Schor, 2005; Knapen & van Ee, 2006). The present study instead focuses on how slant adaptation in one sense (here the haptic sense) can be influenced or interfered with by the availability of information of another sense (here vision) without that information necessarily directly being informative about the slant.

Previous studies have gained insights into the processing level at which haptic adaptation occurs by investigating to which conditions the adaptation aftereffect transfers. For instance, studies have found that haptic adaptation using static contact with a surface (e.g., by placing two fingers or a hand on a surface to estimate the slant or curvature) is largely posture based (Vogels, Kappers, & Koenderink 1996; van Dam et al., 2016) and does not transfer to exploration modes that have a dynamic component such as when moving the finger across the surface (van Dam et al., 2016). This indicates that at least part of the adaptation occurs at the level of the proprioceptors. Such specificity of the exploration mode used for haptic adaptation has been shown for both unimanual (van Dam et al., 2016) and bimanual adaptation when the index fingers of both the left and right hand are used instead of multiple fingers of the same hand (Glowania et al., 2020). Moreover, Glowania et al. (2020) found that these aftereffects were independent of whether an actual object was touched during adaptation or not. That is, slant adaptation aftereffects were observed when simply holding the index fingers of the left and right hand at different prespecified heights in midair during the adaptation phase, before touching a test surface to measure the aftereffect. These findings suggest that we must be adapting constantly to posture rather than haptic shape, regardless of whether we touch something or not. This posture adaptation then influences the perceived shape of subsequently haptically explored surfaces. In our daily life, however, we are hardly aware of the effects of haptic adaptation. This is very surprising, because haptic adaptation aftereffects can already be measured after only two seconds of touching a surface (Vogels et al., 1996). So, either we are not aware of haptic adaptation aftereffects, which is plausible because we also constantly adapt visually without hardly ever noticing it, or we do not adapt in daily life circumstances, perhaps due to additional information from other sensory modalities.

It is important to note that, in all the studies discussed herein, the participants adapted haptically without being able to see the stimulus. This leads to the question if seeing the object, i.e., in the present case, seeing the slant in combination with our finger positions influences haptic adaptation. From previous studies we know, for instance, that, for spatial dimensions such as slant, the sense of vision under normal viewing conditions is typically dominant to the sense of haptics and proprioception because of its greater reliability for such typical viewing conditions (e.g., Rock & Victor, 1964; Ernst & Banks, 2002; van Beers, Wolpert, & Haggard,2002). A study by Kennett, Taylor-Clarke, & Haggard (2001) moreover showed that, for a two-point discrimination task, the threshold for detecting being touched by two stimuli instead of one was significantly lower if the forearm was visible just before stimulation. This result shows that vision can enhance touch. On the one hand, this finding could mean that vision might be partially overruling the haptic adaptation aftereffects when the two are integrated, thus leading to a reduced haptic aftereffect. On the other hand, it has to be noted that the visual modality usually also adapts when presented with the same stimulus for a longer period of time, and it has been shown that unimodal sensory adaptation can transfer between vision and touch (Konkle et al., 2009; Krystallidou & Thompson, 2016). This could mean that, when adapting visually and haptically to the same stimulus at the same time, this leads to an enhanced haptic adaptation aftereffect rather than reducing it, when the visual and haptic aftereffects are added together. The latter hypothesized effect would, most likely require visual fixation for several seconds because of the retinotopic nature of many visual shape-related aftereffects (Knapen et al., 2010; Mathôt & Theeuwes, 2013). In general, however, when exploring a visual scene, we move our eyes at a rate of roughly 1 to 2 saccades per second depending also on the task and thus visually we often tend to not fixate long enough for visual aftereffects to reach awareness. That is, in viewing circumstances that don't require strict fixation we can assume vision to adapt very little. Thus, if there is visual dominance when vision and haptics are integrated, this would provide an explanation for the fact that we do not feel any effects of haptic adaptation in our everyday life as vision may be overruling haptic adaptation.

A recent study by Seizova-Cajic and Azzi (2011) provides some evidence that it may indeed be the case that vision can overrule or cancel touch, or in their case rather proprioceptive, adaptation aftereffects. Their study investigated the effect vision has on the illusionary movement induced when muscles are stimulated with vibration. In their study, the participants’ biceps were stimulated with a strong vibratory stimulus, resulting in an illusionary movement of the arm. Importantly, when the vibration stops this technique also results in perceptual adaptation aftereffects, i.e., perceived movement of the forearm in the opposite direction. Seizova-Cajic and Azzi (2011) found that the strength of the perceived movement for the aftereffect depended on whether participants had seen their arm during the adaptation phase or not. When the participants could see their stimulated arm during adaptation, both the perceived illusory movement effect during the adaptation phase as well as the aftereffect after adaptation were considerably reduced. This finding is consistent with previous work by Lackner and Taublieb (1984), who proposed that vision reduces the effect of the illusionary limb movement from muscle vibration as soon as it is available. However, in their study they found that just seeing the finger or hand was not as effective as seeing the hand or finger and having external objects as a reference. In their study, they had a condition in which the experiment was performed in the dark and one condition in which the experiment was done with the room lights on. In the dark condition, participants wore gloves that were either completely coated in phosphorescent paint or with just the index finger covered in the paint. In this way, the participants could see either their whole hand or just one finger in otherwise complete darkness. Lackner and Taublieb (1984) found that the illusionary movement was much more reduced when the room lights were switched on, i.e., when the hand or finger could be seen in relation to external objects when compared with either of the two conditions in the dark. Thus, it seems that vision influences haptic adaptation only when the visual input is informative about one's own position in relation to external objects. However, in their study only the effect of direct muscle-vibration stimulation was measured without looking further into its aftereffects.

In the present study, we extend these previous findings by investigating in which circumstances and to what extent vision may influence bimanual haptic shape adaptation. In haptic shape adaptation, we can provide visual information not only about our own hand, but also about the touched object and it is not yet clear how these separate elements might each contribute to the visual influence on haptic adaptation. However, given that previous work indicated that haptic slant adaption is largely of a proprioceptive nature (van Dam et al., 2016; Glowania et al., 2020) we were particularly interested in the role of the visual representation of the fingers. To investigate such potential influences from vision, we used the bimanual haptic slant adaptation paradigm as presented in one of our previous studies (Glowania et al., 2020) in which participants used the index fingers of both hands to adapt to a haptic surface slant. We investigated the influence of vision on haptic adaptation by manipulating the kind and number of visual cues presented to the participants during the adaptation phase (e.g., cues about the space only, fingers only, and various combinations). To measure the size of the resulting haptic aftereffect for each condition, we measured the slant at which the surface felt level in the absence of visual information before and after adaptation. Based on the results by Seizova-Cajic and Azzi (2011) and Lackner and Taublieb (1984), we expected a reduction of haptic adaptation when the visual information participants were presented with was informative about the position of the fingertips. Hence, in this study we investigated in two experiments to what extent and in what way visual information about the touched object may also interfere with the adaptation process.

## Methods for experiment 1

### Participants

Twelve members of the Applied Cognitive Psychology Group of Ulm University, three students of Ulm University, and author C.G. participated in this study (16 participants in total, average age 27.9 ± 5.9 years, 5 male). All participants were self-reported to be right handed. They gave informed consent prior to the start of the experiment and the students received 7€ per hour as compensation for their participation in the experiment. Ethical approval was obtained from the Bielefeld University ethics committee.

### Setup

**Figure 1.**
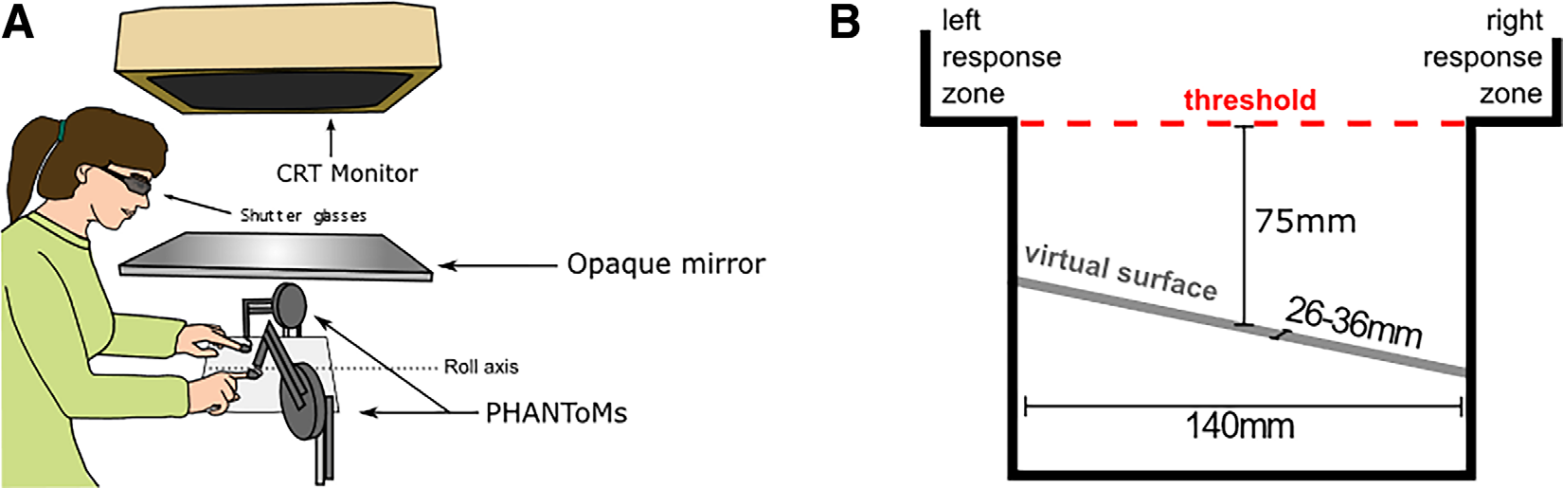
Experimental setup. (**A**) The visuohaptic workbench. The participant was seated in front of the workbench with the body midline aligned with the center of the workbench. Both index fingers were fixed in thimble-like holders attached to the PHANToMs. The visual stimuli were presented on the CRT and viewed via mirror. The system was calibrated to easily present objects both visually and haptically in a spatially aligned way. (**B**) Front view of the virtual space showing the response zones, the threshold for starting a trial and the virtual surface.

Participants were seated in front of a visual-haptic workbench. The workbench consisted of two PHANToM force-feedback devices (PHANToM premium 1.5, SensAble Technologies Inc., Woburn, MA) and a CRT Monitor (Sony CPD G500/G500J, Sony Europe Limited, Weybridge, UK), which was viewed via a mirror in the setup, for visual presentation (Figure 1A). The PHANToM force-feedback devices were placed one to each side of the workbench for haptic presentation of the stimulus. The index fingers of the participants were fixed with rubber bands in thimble-like holders at the end of the force feedback devices. These devices were used to create virtual surfaces by generating independent forces to the fingers. This way, the participants could feel and interact with virtual haptic surfaces in an automated setting. Direct vision of the hands was prevented. Visual feedback of the finger position was only available when needed experimentally, in which case it was presented stereoscopically on the CRT monitor. The participants placed their chin on a chinrest to keep their body midline aligned with the center of the workbench and to allow accurate rendering of the visual cues. To present the visual stimulus in three-dimensional (3D) stereoscopic depth, 3D-shutter glasses (RealD Pro CrystalEyes 4S VRLOGIC GmbH, Dieburg, Germany) were used to present the visual scene for the left and right eye separately. The stimuli for this experiment were created using a custom build program in C++, GHOST (for the haptic display) and OpenGL (for the visual display).

### Stimuli

The stimuli used in this study consisted of several different components, each of which could either be present or absent during the adaptation phase. Note, however, that during test phases only a haptic surface was presented as the test stimulus was kept the same across all adaptation conditions. The haptic surface was rendered by the PHANToM force feedback devices and could be presented at different angles of slant (Figure 1). The angle of the surface, if present during adaptation, was kept consistent during the (top-up) adaptation phases at ±10 deg slant, but on test trials could vary according to a 1-up/1-down staircase procedure. The exploration of the haptically rendered surface was limited to 140 mm left to right and 26 mm in depth.

If the surface was also visually presented during the adaptation phase, the visual surface would have the same surface slant (±10 deg slant). However, visually the surface was presented at a slightly larger scale to avoid potential conflicts at the surface edges. The visual surface extended either 145 mm (when combined with haptic elements) or 175 mm (when presented in isolation) in length and 36 mm in depth. The slightly shorter visual surface length when combined with haptic elements (i.e., 145 mm vs. 175 mm) was chosen to avoid the participants going toward the edges of the haptic space in the bimodal cases.

For conditions that included cursors indicating the positions of the fingertips, the Visual Cursors were visually rendered spheres (10 mm in diameter). These spheres were used to give feedback about the position of the participants’ fingers, without participants being able to see their own fingers directly.

**Figure 2.**
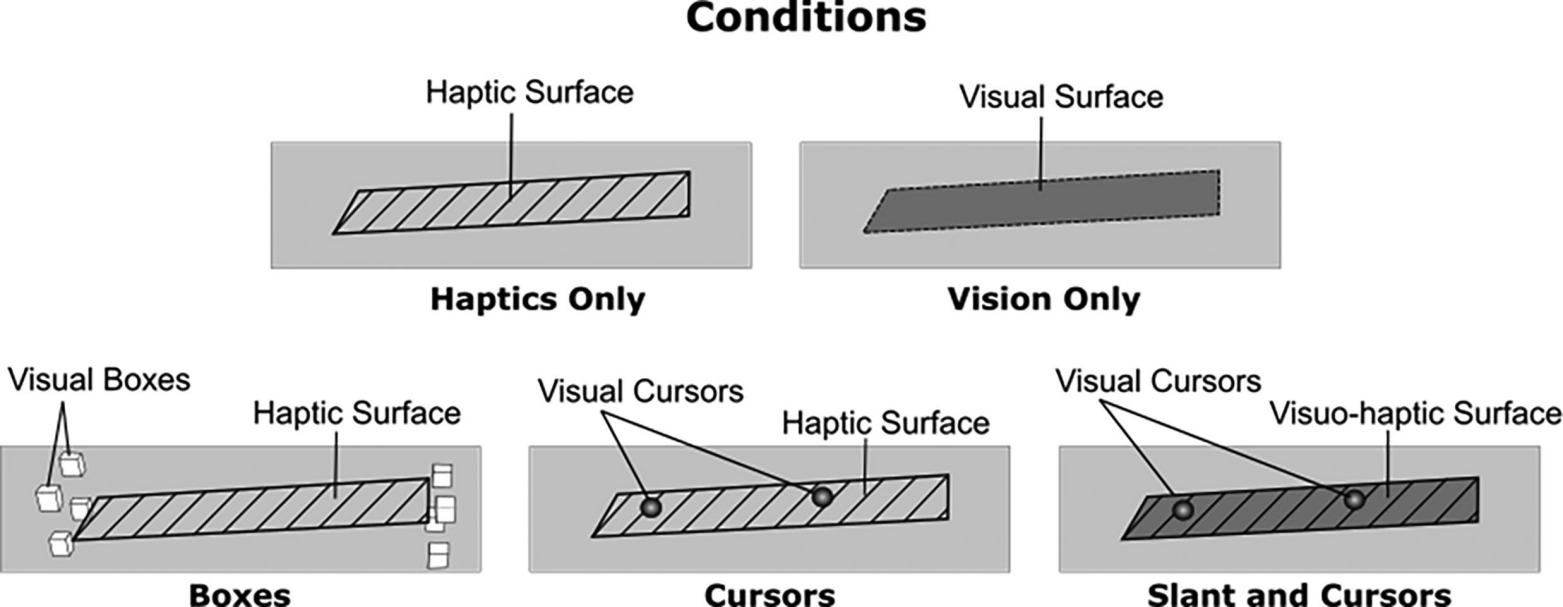
Adaptation conditions. Haptics Only: Subjects could feel the slanted surface but no visual cues were presented. Vision only: Subjects could see the slanted surface but not touch it. Boxes: Subjects could see eight boxes framing the area in which the haptic surface was presented; however, the slanted surface itself could only be felt but not seen. Cursors: Subjects could see the position of their index fingers as cursors, but the slanted surface was only rendered haptically. Slant and cursors: Subjects could see the position of their index fingers as cursors as well as the surface slant, which was also rendered haptically.

In some conditions, a visual spatial reference was provided by adding what we will call Visual Boxes into the visual scene. These Visual Boxes were eight reference boxes (size: 10 mm × 10 mm × 10 mm) positioned 100 mm left and right of the body midline, 25 mm above and below the surface center, and 13 mm to the front and back from the surface midline (Figure 2, bottom left). The Visual Boxes were used to provide a visual spatial reference, without necessarily providing direct information about either the slant or the location of the participant's fingertips. Such a stable reference frame can however potentially be relevant to help interpret the visual location of other visual elements (i.e., cursors or a slanted surface) more accurately (see Lackner & Taublieb, 1984).

### Adaptation conditions

The elements described above were used in five adaptation conditions, which we explain in this section. The conditions can be divided into unimodal and bimodal adaptation conditions. In the unimodal adaptation conditions the surface was either presented only visually (Vision Only condition) or only haptically (Haptics Only condition) (see Figure 2, top row) and served as baseline conditions to which the bimodal conditions were compared. The bimodal conditions were different combinations of vision and haptics (see Figure 2, bottom row).

### Condition: Haptics Only (unimodal adaptation)

In the Haptics Only condition (Figure 2, top left), only the haptic surface was presented. This means that the screen was black and no visual cues were presented to the participant, but participants were able to touch the slanted surface. Hence, this condition is a unimodal haptic condition and served as the haptic baseline condition to measure the haptic aftereffect without any potential interaction from vision.

### Condition: Vision Only (unimodal condition)

In the Vision Only condition (Figure 2, top right), only the visual surface, was presented. This means that participants viewed the slanted surface during the adaptation phase, but the surface was not rendered haptically and, thus, participants were not able to touch the surface. This condition can be considered as the unimodal visual condition and served as the visual baseline condition to measure to what extent a visual slant aftereffect can transfer to haptics (because the test phase was always haptic only).

### Condition: Boxes (bimodal condition)

In the Boxes condition (Figure 2, bottom left), participants were able to touch the slanted surface haptically and furthermore view eight boxes, rendered always in the same position irrespective of the slant of the haptic surface or the position of the fingers. Thus, these boxes served merely as a visual spatial reference for the general location of the haptic space, without providing any slant or finger information. Since both visual and haptic information was provided, this condition is considered a bimodal condition. Note, however, that, despite both visual and haptic information being available in the general display, no single element within the scene was truly bimodal. This condition, thus, served as a bimodal control for slant adaptation, to verify whether or not the information in the two modalities needs to match.

### Condition: Cursors (bimodal condition)

In the cursors condition (Figure 2, bottom center), the participants could touch the slanted surface haptically and, furthermore, could see two little spheres corresponding in location to the position of their fingertips (i.e., the cursors). These cursor positions also contained some limited visual information about the slant of the surface because participants could visually compare the heights of the fingertips while touching the surface. Note that, in this case, the positions of the fingertips present actual bimodal information, and, using that information, the surface slant can be sensed as well through both proprioception and vision simultaneously. This condition is informative about whether visual information of the fingertips is sufficient to interfere with haptic slant adaptation.

### Condition: Slant and Cursors (bimodal condition)

In the Slant and Cursors condition (Figure 2, bottom right), the participants were able to see as well as touch the slanted surface, and additionally the cursors representing the participant's fingertips were presented. This means that participants could also relate the visual position of the fingertips to the visuohaptic surface. Because visual and haptic information of both the slanted surface and the positions of the fingertips were presented congruently, this condition is considered bimodal and both the slanted surface and the fingers are both bimodal features.

### Procedure

Each participant conducted each of the five adaptation conditions in a separate session, with a break of at least 10 minutes between sessions. Breaks were introduced to minimize carry-over effects. Because Vogels, Kappers, and Koenderink (1995) showed that the haptic aftereffect of a curved surface decays quickly and is hardly present after 80 Seconds, we inferred that 10 minutes should be more than enough for the haptic aftereffect to vanish. The order of the conditions was randomized across participants.

For each of the adaptation conditions, we followed a “pre-test–adaptation–post-test” paradigm. Test trials in the pre- and post-tests testing for haptic aftereffects were identical for all five conditions and only contained a haptically rendered surface without visual cues. For test trials, the participant's task was to decide whether the surface was tilted to the left or to the right, that is, to answer the question “Which side of the surface was higher: left or right?” In order to calculate the effect of adaptation on haptic slant perception, we used the participant's answers to these test-trials determining the point at which the participants haptically perceived the surface slant as level—that is, the Point of Subjective Equality (PSE)—before adaptation (in the Pre-Test) and after adaptation (in the Post-Test). To present the trials for the PSE measurements, we used a 1-up/1-down staircase procedure (for more information, see, e.g., Gescheider, 1976), which converges on the point at which participants respond left side higher and right side higher with equal proportions (i.e., the PSE). The step size between test trials started with 8 deg, after two reversals in responses in the staircase procedure, the step size was decreased to 4 deg and after another two reversals to 2 deg. After 12 reversals, the staircase was terminated. To avoid potential hysteresis effects, we presented two intermixed staircases that started at different angles, i.e., one staircase started at –20 deg, while the other started at +20 deg. To calculate the PSE for each condition, we fitted psychometric curves (cumulative Gaussians) to the combined data from the two staircases for each test-phase, resulting in one PSE for the Pre-Test before adaptation and Post-Test after adaptation, respectively. We chose this method for obtaining the PSE, rather than, for example, averaging over reversals points in the staircase, because it allows us to use the data from all trials. For fitting the psychometric curves, we used the psignifit toolbox for MATLAB version 2.5.6 (Wichmann & Hill, 2001a; Wichmann & Hill, 2001b), with mu (mean) and sigma (standard deviation) as free parameters for the cumulative Gaussian. The lapse-rate was fixed to zero, which given symmetry between left and right side higher stimuli/responses on either side the PSE, should not affect the positioning of the PSE. The PSE on the psychometric curve is the only point of interest in our particular case as 1-up/1-down staircases are not well-suited to determine the slope or the JND because of the sparse sampling away from the PSE. The PSE per test-phase was determined by obtaining the 50% cutoff of the psychometric curve (i.e., equal proportion of left side higher and right side higher responses). The size of the aftereffect was then calculated by subtracting the post-test PSE from the pre-test PSE.

To familiarize participants with the task, they received as many practice test-trials as they liked at the start of each experimental session. As soon as both participant and experimenter were satisfied that the task was understood, the experimental procedure started.

### Pre-test

Each trial in the pre-test started by showing the trial number on the screen. The participants lifted both of their index fingers until they reached a predefined threshold (75 mm above the surface, see Figure 1B) and the trial number disappeared. Next, participants lowered their left and right index fingers until they reached the haptic surface. The participants were instructed to keep the fingers in static contact with the surface, that is, without actively moving the fingers over the surface. The surface was haptically rendered for 1 second as soon as one of the fingers touched the surface. After that 1 second for estimating the slant, the surface disappeared. Next, participants responded which side of the previously felt surface appeared higher: left or right. Participants made their response by moving with either the left or right finger into the corresponding response zone located at the top, left (left side higher response) and right (right side higher response) of the exploration space (Figure 1C). After providing a response, the next trial number appeared on the screen and the next trial started. Trials continued until the staircases terminated after 12 reversals.

### Adaptation

Following the pre-test, the main adaptation phase started. Similar to the pre-test, participants started the main adaptation phase by raising their fingers until they crossed the trial-start threshold. During this phase, depending on the adaptation condition, participants could either see only: 1) the slant (Vision Only), 2) nothing (Haptics Only), 3) eight boxes (Boxes), 4) their own fingertips as cursors (Cursors), or 5) a combination of cursors and slant (Slant and Cursors). As before, participants lowered their fingers until they reached the adaptation surface (during adaptation this was always a ±10 deg slant) and held static contact with the surface for the whole adaptation period of 30 seconds. Note that the direction of the adaptation slant was counterbalanced between participants. As soon as one finger touched the surface, the 30 seconds of the adaptation phase started. This was the case for all conditions, except the Vision Only adaptation condition. In the Vision Only condition, since there was no haptic surface rendered, the display time started as soon as the threshold (the 75-mm threshold) (Figure 1B) for starting the trial was crossed by the participant's fingers during the downward movement. Furthermore, for this condition the participants were instructed to hold their fingers in the air during the adaptation phases. After the main adaptation phase ended the haptic surface (if rendered) as well as the visual scene (if present) disappeared. For the main adaptation phase, the participants were not asked to judge the surface slant and after the 30 seconds of adaptation, the program automatically proceeded to the post-test phase.

### Post-test

The procedure of the post-test was the same as in pre-test, with the only difference that a 4 seconds top-up adaptation interval preceded each test trial in the post-test. After the 4 seconds were up, the test trial immediately started. During the top-up adaptation interval, the same visuohaptic condition (Haptics Only, Vision Only, Boxes, Cursors, Slant and Cursors) was presented as in the main adaptation phase. Similar to the main adaptation, the participants did not need to judge the surface slant for top-up adaptation intervals. The task of judging the slant only needed to be performed for the actual test-trials. Note, that for test trials in the post-test the surface slant was again presented only haptically and no visual information was shown. Both staircases were again terminated after 12 reversals each to complete the session.

## Results of experiment 1

**Figure 3.**
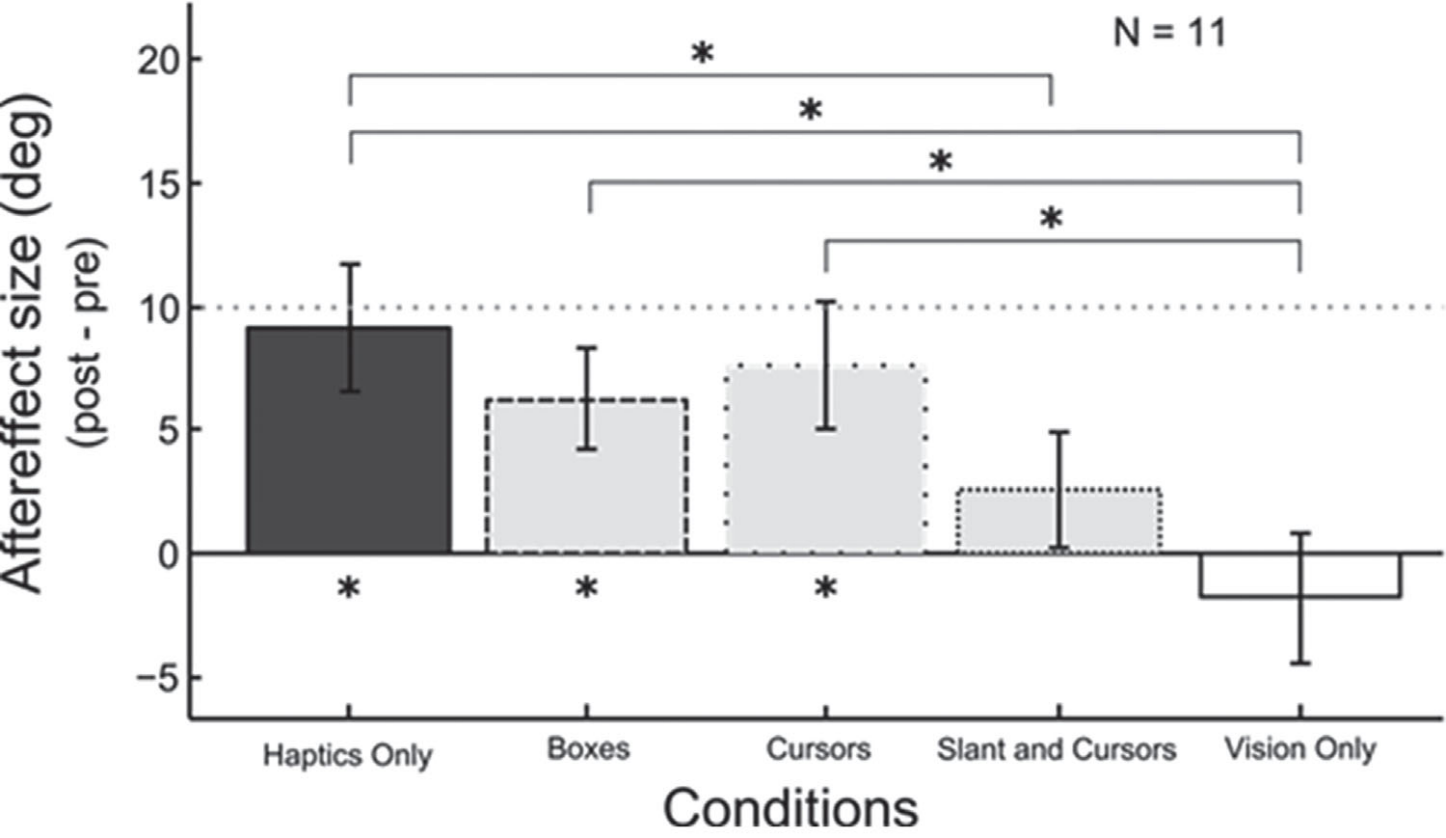
Results of experiment 1. The *y* axis shows the haptic adaptation aftereffects as a result of different visual and haptic adaptation conditions. Each bar represents a different adaptation condition as indicated on the *x* axis. Error bars are standard errors. The dotted line marks the point at which full adaptation would occur. Note that, on the test trials for all adaptation conditions, the surface was rendered only haptically.

The results of five participants (all female, right handed) were removed from the data analysis because their staircases did not converge within 40 trials in at least one of the conditions. Data from the remaining 11 participants were analyzed. To investigate the influence of vision on haptic adaptation, participants adapted to different conditions in which the amount of visual information about the slant varied. Besides two unimodal baseline conditions (Vision Only and Haptics Only rendering of the surface), participants were presented with three bimodal adaptation conditions in which, besides the haptic surface, participants either received only visual information about their fingers position (Cursors), or about the space in which the surface was rendered (Boxes), or about the slant and the fingers position (Slant and Cursors). Figure 3 shows the sizes of the aftereffects derived from all five adaptation conditions. First, the two baseline conditions—Haptics Only (the left-most bar) and Vision Only (right-most bar)—reveal very different haptic aftereffects: strong adaptation for the Haptics Only condition and no haptic adaptation for the Vision Only condition. This indicates that presenting only a visual but no haptic surface as an adapter stimulus does not lead to any noticeable haptic slant aftereffect. Note that this does not mean that the visual slant interfered with haptic adaptation in this case, as the Vision Only condition is the only condition in which, during adaptation, no haptic surface was presented and thus no haptic adaptation to interfere with. Rather, this result suggests that adaptation to visual slant does not necessarily transfer into a haptic adaptation aftereffect upon testing. These two baseline conditions furthermore frame the bimodal conditions for comparison.

To test which conditions resulted in significant haptic slant aftereffects, we performed one sample *t* tests against zero for each condition. A Bonferroni correction was applied by using an alpha of 0.01. The tests revealed that of the two unimodal adaptation conditions only the adaptation condition in which the haptically rendered surface was present as adapter stimulus (Haptics Only) was significantly different from zero (*t*(10) = 4.6, *p* < 0.001), whereas the Vision Only was not (*t*(10) = −0.9, *p* = 0.409). For the bimodal conditions, the *t* tests against zero revealed that the results for the Boxes as well as the Cursors conditions are significantly different form zero (Boxes: *t*(10) = 5.1, *p* < 0.001; Cursors: *t*(10) = 3.7, *p* = 0.004), whereas the condition in which the slant as well as the cursors were visually presented was not (*t*(10) = 1.5, *p* = 0.157).

To test for significant differences between the conditions, we first performed a one-way repeated measures ANOVA, which was significant (*F*(4,40) = 5.4, *p* = 0.001). We then investigated where the significance came from by performing paired sample *t* tests between the conditions. If vision influences haptic adaptation, we expect a significant reduction of the aftereffect in the conditions containing visual feedback compared with the Haptics Only condition.

The *t* tests revealed that the two unimodal conditions haptics only and vision only were significantly different from each other (*t*(10) = −4.0, *p* = 0.002). Furthermore, the haptics only condition showed a significant difference when compared with the slant and cursor condition (*t*(10) = −2.4, *p* = 0.040). When compared with the unimodal vision only condition, significant differences were found for the bimodal conditions boxes and cursors, boxes vs. vision only: *t*(10) = −3.9, *p* = 0.003; cursors vs. vision only: *t*(10) = 2.6, *p* = 0.027. Only the bimodal slant and *Cursor* condition was not significantly different from the vision only condition (*t*(10) = 1.8, *p* = 0.110).

## Discussion of experiment 1

In Experiment 1, we tested if visual information impacts the haptic adaptation to slant. To this end, we varied the amount of visual information available to the participant by having conditions in which only one sensory modality was available to participants (Haptics Only, Vision Only) and conditions in which the two modalities were combined. In the conditions with combined modalities, the haptic information was always the same but the type of visual information available was varied (Cursors, Boxes, Slant and Cursors conditions).

First, the result of the Haptics Only condition is significantly different from zero, showing that adaptation to slant occurs in conditions in which the information from both hands is needed for estimating the slant (bimanual adaptation). This confirms the results from our previous study that focused on the level at which bimanual slant adaptation occurs (Glowania et al., 2020). Furthermore, the Vision Only condition was not significantly different from zero, suggesting that pure visual adaptation does not lead to an aftereffect in a purely haptic test condition (no transfer). That is, when only visual but no haptic slant information is provided during the adaptation phase, there seems to be no measurable effect when using the testing condition in which only a haptic surface was presented.

Next, we looked at how different visual cues during adaptation affected haptic slant adaptation. We found that the condition in which two visual cues were available, i.e., the Slant and Cursors condition, had a significantly reduced haptic aftereffect compared with the unimodal Haptics Only condition. This result is a strong indicator that vision does reduce haptic/proprioceptive adaptation to slant and extends the findings by Seizova-Cajic and Azzi (2011) as well as those of Lackner and Taublieb (1984), in relation to proprioceptive muscle vibrations, to the case of haptic shape adaptation. In contrast, the two conditions in which only one visual cue was available, that is, the Boxes condition and the Cursors condition, the aftereffect was not significantly different from the condition in which only haptic information was presented. Thus, we did not find a significant reduction of the slant aftereffect in this case. Based on these findings, it seems that the more visual cues are available to the participant during adaptation, the less strong the adaptation aftereffect in the haptic domain is.

To further test the hypothesis that the combination of visual cues is important for the influence on haptic adaptation, we conducted a second experiment. Note, that in the first experiment the Boxes condition and the Cursors condition alone where not enough to prevent the participants from adapting in the haptic domain. Thus, the question arises as to whether the combination of the two would lead to a reduced aftereffect. Like the visual slant in the Slant and Cursors condition, the boxes could provide a reference cue for the spatial position of the cursors (which in turn can be linked to the proprioceptive estimate of the finger positions). More particularly, however, the Slant and Cursors condition allowed participants to directly link the finger positions to the actual slant of the surface within a single frame of reference across the senses. That is, the visual slant provided both direct visual information about the surface slant, as well as a more general spatial reference in which to interpret the cursors. In a combination of the boxes and the cursors, the link to the surface slant is much less direct, but the boxes could still act as a spatial anchor or reference for the cursors. Hence, if we find a reduced aftereffect when the boxes and the cursors are combined, this would mean that providing a spatial reference to the cursors already reduces the size of the aftereffect, meaning that a direct link between slant and cursors is not needed for a reduction of the aftereffect. If the combination of the boxes and cursors does not result in a reduced aftereffect, however, this would lead to the conclusion that directly seeing and linking the touched surface to the fingers’ position is crucial for reducing the adaptation aftereffect.

## Methods for experiment 2

### Participants

In total, 11 students of Bielefeld University (average age 24.5 years, 3 male) participated in this study. Ten participants self-reported to be right handed; one was left handed. They gave informed consent before the start of the experiment and received 6€ per hour as compensation for their participation. Ethical approval was obtained from the Bielefeld University ethics committee.

### Setup and procedure

**Figure 4.**
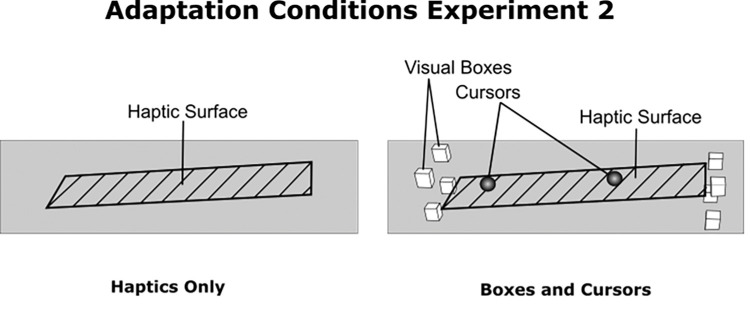
The conditions of experiment 2. Haptics Only: The subjects could only feel the surface, but no visual information was available. Boxes and Cursors: Both the cursors representing the position of the fingertips as well the 8 visual boxes that spanned the available exploration space were presented (for details, see descriptions for experiment 1). The surface itself was not visually represented, but was available for haptic touch.

The experimental setup was the same as for experiment 1. For the procedure, we reduced the number of reversals for the staircase procedure to eight reversals, since the analysis of the results from experiment 1 showed that generally the staircases had already fully converged after this number. Furthermore, we reduced the number of conditions to two: Haptics Only and Boxes and Cursors. The Haptics Only condition was the same as in the previous experiment and the surface could be felt but no visual cues were provided. The condition Boxes and Cursors was a mixture of the Boxes and the Cursors conditions of experiment 1 (Figure 4, right). This means that the participants could see the positions of their fingers as cursors but additionally saw the eight boxes providing a spatial reference (see Materials and Methods for Experiment 1 for details). In both conditions, the slanted surface itself was rendered only haptically and thus the participants received haptic feedback when touching the surface without seeing the actual surface.

## Results of experiment 2

**Figure 5.**
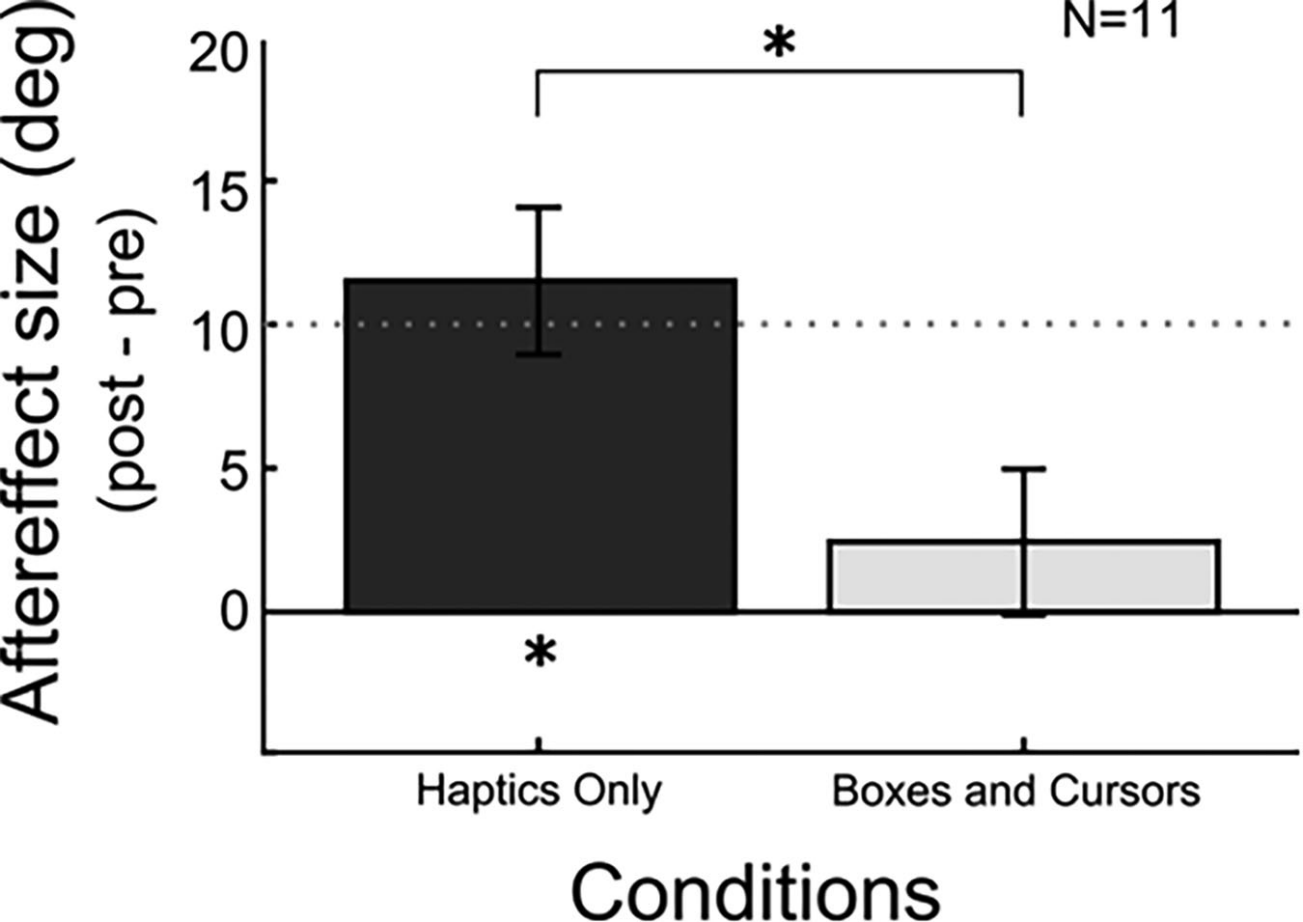
Results of experiment 2. The *x* axis shows the labels for each condition, the *y* axis the size of the aftereffect, and error bars are the standard error. The dotted line marks the point at which full adaptation would occur.

To investigate the influence of vision on haptic adaptation, participants adapted to a slanted surface when vision was not available (Haptics Only) and to a slanted surface when they could see the location of their fingertips represented as spherical cursors as well as eight reference boxes providing a visual reference frame. Figure 5 shows a significant aftereffect for the Haptics Only condition (*One-sample t* test: *t*(10) = 7.7, *p* < 0.001) of comparable magnitude to experiment 1. However, there was no significant effect for the Boxes and Cursors condition (one-sample *t* test: *t*(10) = 1.2, *p* = 0.252). Furthermore, there is a significant difference between the Haptics Only condition and the Boxes *and* Cursors condition (*paired t* test: *t*(10) = −4.0, *p* = 0.002).

In addition, we also statistically compared the results of the two experiments using an independent samples *t* tests. Those confirmed that there are no significant differences between the Haptics Only conditions from experiments 1 and 2 (independent samples *t* test: *t*(20) = 0.96, *p* = 0.351), and between the Boxes and Cursors condition of the second experiment and the Slant and Cursors condition of the first experiment (Indipendent *t* test: *t*(20) = 0.04, *p* = 0.965).

## Discussion of experiment 2

In the second experiment, we tested whether providing the boxes, instead of the surface slant (the Slant and Cursors condition of experiment 1) as a second visual cue as well as the cursors, would lead to the same reduction of haptic adaptation aftereffects. First, as in experiment 1, we found a significant aftereffect in the Haptics Only condition, showing again that without visual information haptic adaptation to slanted surfaces occurs. Moreover, the results of experiment 2 showed a significant reduction of the aftereffect in the Boxes and Cursors condition when compared with the Haptics Only condition and in this case the aftereffect was also not significantly different from zero.

When comparing the results of both experiments, no significant differences were found between the two Haptics Only conditions (from experiments 1 and 2) as well as between the two conditions containing visual feedback about the fingertips’ positions (cursors) and a spatial reference (slant for experiment 1 and boxes for experiment 2). This process reveals that a visual spatial reference is relevant in combination with the cursors to see the interference with haptic adaptation, but that the type of reference does not seem to play a greater role.

The results of experiment 2 indicate that the visual information given by the cursors in combination with reference boxes is enough information to prevent the participants from haptically adapting to the surface slant. Interestingly neither the cursors nor the boxes by themselves provided enough information to reduce haptic adaptation as shown in the results of experiment 1. These findings suggest that spatially linking the visual cursors, that is, giving the visual cursors a spatial context, suffices to reduce the size of the haptic aftereffect. Thus, as the results of experiment 2 show, it is not even necessary to provide visual information about the actual adaptation surface (slant) itself.

## General discussion

In this study, we investigated how visual information can affect haptic slant adaptation. The results of both experiments show that the availability of visual information does reduce haptic adaptation. However, the results of experiment 1 showed that making just any visual cue available while haptically adapting to slant is not enough to reduce haptic adaptation. This result was most apparent for the condition in which either only the cursors or only the reference boxes were visually presented. In these cases, haptic adaptation was not significantly reduced compared with the Haptics Only condition. It seems that, for vision to affect haptic adaptation, there needs to be a meaningful visual spatial context in which the position of the fingers can be understood. For instance, by the combination of a visual representation of the touched slant and the cursors (Slant and Cursors condition of experiment 1), vision provides information about the position of the fingertips as well as a spatial reference of the space. Interestingly the visual spatial reference did not need to include direct information about the slanted surface, but it sufficed to show reference boxes spanning the area in which the slant was presented as in the Boxes and Cursors condition of Experiment 2.

Note that our results can be interpreted as being in conflict with a study by Konkle, Wang, Hayward, and Moore, (2009). Konkle et al. (2009) found that motion adaptation aftereffects transfer between vision and touch. Based on this finding, one might expect that, when the surface was seen but not felt (Vision Only condition), any visual adaptation should transfer to the test condition in which only haptic cues were available and no visual information was shown. In the present study we, however, did not find a haptic slant adaptation aftereffect when the surface was presented only visually during adaptation, although it is known that visual slant is subject to visual adaptation (e.g., Köhler & Emery, 1947; Wenderoth, 1970; Adams, Banks, & van Ee, 2001). There are several possible explanations for this discrepancy. First, we did not measure the visual aftereffects of adaptation and, therefore, can only speculate about a possible existence of such effects in the present task. On the one hand, visual adaptation to slant has been shown to occur for both stereo disparity and perspective cues (e.g., Bergman & Gibson, 1959; Knapen & van Ee, 2006). Thus, we have no reason to believe that the visual system did not adapt in the present case. On the other hand, visual adaptation can be retinal location specific and thus needs fixation rather than free eye movements. Even under strict fixation some adaptation effect might not be complete as microsaccades have been shown to prevent certain types of visual adaptation (e.g., Martinez-Conde, Macknik, Troncoso, & Dyar, 2006; Habtegiorgis, Rifai, Lappe, & Wahl, 2017). In the present study, however, participants were free to make eye movements and it is likely that the participants moved their eyes across the visual scene. This could potentially lead to less adaptation of the visual system. Second, in the study by Konkle et al. (2009), haptic motion judgments were based on cutaneous cues whereas we used PHANToM force-feedback devices in which only proprioceptive cues were available. Because there is evidence that cutaneous and visual receptive fields overlap in the brain (e.g., see Graziano & Gross, 1993; Graziano & Gross, 1995), the processing of these types of information could potentially be more strongly linked. In our case, however, haptic slant estimates were obtained by comparing the finger positions of the left and right hands (see also Glowania et al., 2020), and thus the estimate with regard to the seen slant of the actual object is indirect at best and more of proprioceptive nature rather than cutaneous. Since in this case the low-level sensations (haptic finger positions and visual object slant) do not directly correspond to the same estimate, this can explain why we do not see any transfer from visual slant to the haptic domain.

In light of the above and to interpret the present results, it is meaningful to distinguish between the elements that were truly bimodal (i.e., both visually and haptically represented) and those that were not. Here, it is important to remember that the cursors provide information about the position of the fingers that can also be directly sensed through proprioception. Therefore, it is most likely the cursors, rather than, for instance, the surface slant, that were truly bimodal elements in the present task. Because of the cursors, there is a direct link of the proprioceptive input of the fingertips to the visual input about fingertip position. Furthermore, the seen movement on the screen, especially at the beginning of the trial when the participant lowers the fingers until the haptically rendered surface is reached, is in the temporal alignment of the participants’ finger movement. This temporal alignment does not only lead to optimal integration of the visual and haptic information (Plaisier, van Dam, Glowania, & Ernst, 2014), but should also lead to a sense of agency, because what the participants see is in alignment with what they do (Haggard & Chambon, 2012). In all the conditions in which the cursors are not present, this direct link between the visual and the haptic input is missing and did not reduce the haptic aftereffect. Still, seeing only the finger positions through the cursors did not result in a significantly lower aftereffect either. This is because the cursors alone do not necessarily provide all that much information about their position in 3D space. Visual depth perception is known to rely more on relative depth cues (e.g., relative disparity) (Erkelens & Collewijn, 1985) rather than absolute depth cues (e.g., convergence of the eyes) (Richards & Miller, 1969). When only the cursors are available, there is relatively little relative depth information provided, since, for each cursor, there is only the other cursor (representing the other finger) to compare the visual distance to. Hence, the cursors alone might not give sufficiently reliable visual information to estimate the finger positions visually in 3D space. A second visual cue is needed to provide a relative spatial reference for the cursors, that is, to make the height difference between the visually displayed cursors more obvious. It, however, does not seem to matter whether the reference is itself directly informative about the slant of the surface or not, which is shown in the lack of a significant difference when comparing the Slant and Cursors condition of experiment 1 with the Boxes and Cursors condition in experiment 2. Both, the touched slant as well as simple reference boxes, give the cursors spatial context and result in a significantly reduced haptic adaptation. This is consistent with the results by Lackner and Taublieb (1984), who found that the proprioceptive muscle spindle vibration illusion is much weaker when the own body is seen in context with an external reference.

In short, our results suggest that there may be something special about adding a visual spatial context with respect to which to interpret the positions of the cursors. This addition could be in the form of adding some spatial anchors in the work space that allow to distinguish positions and movements of the cursors more easily and more reliably. If so, it could be of interest to investigate in future work just how the spatial reference might influence the reliability of the visual cursor positions and in turn how this affects haptic slant adaptation. Future studies could, for instance, vary the reliability of the visual reference cues, such as the boxes, and/or the cursors through blurring or similar techniques. If it is the visual relative spacing between cursors and boxes that is relevant for influencing haptic adaptation, then visually degrading either one, thus decreasing the visual reliability of this relative spacing, should lead to haptic aftereffects occurring again to the same extent. Doing the same for a different combination of visual cues without the cursors (e.g., slant and boxes) should not lead to any changes in results. By measuring these reliabilities in separate experiments, these potential relationships could then also be quantified. This was beyond the scope of the present work, however, where we first elucidated the potential relevance of a spatial reference, and we leave the investigation of the relationship between visual reliability of the space and the haptic adaptation aftereffect to future work.

## Conclusions

In the present study, we investigated the influence of different types of visual cues on haptic adaptation. Our results indicate that as soon as visual input provides sufficient information about the position of our fingers in space (i.e., vision provides information about both finger positions as well as their spatial context), haptic adaptation no longer occurs. This might explain why we are not constantly affected by haptic adaptation aftereffects when haptically exploring the world around us.

Our results are of special importance when developing and designing virtual haptic environments. Visual feedback needs to be meaningful and provides a spatial context to the operator to prevent adaptation. Without such a visual reference, adaptation will soon lead to quite substantial inaccuracies in estimated finger/hand positions, which will ultimately prevent operators in such environments from performing their task at an acceptable level.
